# Comparison between Nanoparticle Encapsulation and Surface Loading for Lysosomal Enzyme Replacement Therapy

**DOI:** 10.3390/ijms23074034

**Published:** 2022-04-06

**Authors:** Eameema Muntimadugu, Marcelle Silva-Abreu, Guillem Vives, Maximilian Loeck, Vy Pham, Maria del Moral, Melani Solomon, Silvia Muro

**Affiliations:** 1 Institute for Bioscience and Biotechnology Research, University of Maryland, College Park, MD 20742, USA; emeemapristis@gmail.com (E.M.); vpham197@terpmail.umd.edu (V.P.); melani_solomon@yahoo.com (M.S.); 2 Institute for Bioengineering of Catalonia, Barcelona Institute for Science and Technology, 08028 Barcelona, Spain; marcellesabreu@gmail.com (M.S.-A.); guillev9837@gmail.com (G.V.); mloeck@ibecbarcelona.eu (M.L.); delmoral1999@gmail.com (M.d.M.); 3 Department of Chemical and Biomolecular Engineering, University of Maryland, College Park, MD 20742, USA; 4 Catalan Institution for Research and Advanced Studies, 08010 Barcelona, Spain

**Keywords:** enzyme therapeutics, poly(lactic-co-glycolic acid) nanoparticles, encapsulation, surface loading, ICAM-1 targeting, lysosomal delivery, in vivo biodistribution

## Abstract

Poly(lactide-co-glycolide) (PLGA) nanoparticles (NPs) enhance the delivery of therapeutic enzymes for replacement therapy of lysosomal storage disorders. Previous studies examined NPs encapsulating or coated with enzymes, but these formulations have never been compared. We examined this using hyaluronidase (HAse), deficient in mucopolysaccharidosis IX, and acid sphingomyelinase (ASM), deficient in types A–B Niemann–Pick disease. Initial screening of size, PDI, ζ potential, and loading resulted in the selection of the Lactel II co-polymer vs. Lactel I or Resomer, and Pluronic F68 surfactant vs. PVA or DMAB. Enzyme input and addition of carrier protein were evaluated, rendering NPs having, e.g., 181 nm diameter, 0.15 PDI, −36 mV ζ potential, and 538 HAse molecules encapsulated per NP. Similar NPs were coated with enzyme, which reduced loading (e.g., 292 HAse molecules/NP). NPs were coated with targeting antibodies (> 122 molecules/NP), lyophilized for storage without alterations, and acceptably stable at physiological conditions. NPs were internalized, trafficked to lysosomes, released active enzyme at lysosomal conditions, and targeted both peripheral organs and the brain after i.v. administration in mice. While both formulations enhanced enzyme delivery compared to free enzyme, encapsulating NPs surpassed coated counterparts (18.4- vs. 4.3-fold enhancement in cells and 6.2- vs. 3-fold enhancement in brains), providing guidance for future applications.

## 1. Introduction

Over the last few decades, proteins have emerged as an important class of therapeutics due to their applications in a broad range of diseases, including cancer, autoimmune disorders, rare genetic syndromes, hematological maladies, metabolic conditions, etc. [1,2,3]. The US Food and Drug Administration has approved more than 200 protein therapeutics and hundreds of products are currently under clinical development [4]. Clinically approved protein therapeutics include drugs that either supplement endogenous proteins (cytokines, growth factors, enzymes, or coagulation factors) or block the activity of endogenous proteins (monoclonal antibodies, soluble receptors, or enzyme inhibitors) [5]. However, the potential of protein-based therapeutics is often challenged due to their physicochemical properties, including their high molecular weight, hydrophilicity, and presence of charged functional groups on their surfaces [6,7,8,9]. These properties pose biopharmaceutical challenges such as poor bioavailability, short half-lives, or poor permeability across biological membranes [6,7,8,9]. Proteolytic and enzymatic degradation of protein therapeutics in vivo, immunogenicity due to antigenic determinants on their structure, and non-specific toxicity via unwanted distribution in non-targeted organs represent additional obstacles in the development of protein therapeutics [10,11,12].

In this regard, pharmaceutical nanocarriers provide an excellent alternative to improve the delivery of protein therapeutics by preventing their rapid interaction with serum species, degradation, and immune recognition [13,14,15]. In addition, nanocarrier functionalization with targeting moieties (antibodies, peptides, ligands, aptamers, etc.) can improve the delivery of proteins to specific organs, tissues, or cells [16,17,18]. Numerous drug delivery systems have been investigated for this purpose, including self-assembly in micellar systems, polymeric nanoparticles (NPs), liposomes, carbon nanotubes, metallic NPs, silica NPs, dendrimers, and many others [13,15,19,20,21]. Among these listed nanocarriers, polymer-based NPs hold great potential for protein delivery due to their stability and multifunctionality [22]. Polymeric NPs can be prepared using either biodegradable synthetic polymers such as poly(lactide-co-glycolide) (PLGA) copolymers, polyacrylates, poly(caprolactone)s, polyphosphazenes, or natural polymers such as albumin, gelatin, alginate, collagen, or chitosan [14,22,23,24,25].

A relevant example of therapeutic application based on protein delivery is that of enzyme replacement therapy (ERT), such as the case for the treatment of several lysosomal storage disorders (LSDs). LSDs are a group of about 60 inherited conditions characterized by multi-organ defects [26]. They associate with extensive accumulation of undigested substrates in lysosomes within cells as a result of genetic deficiencies affecting mainly lysosomal enzymes and respective metabolic pathways [26]. ERT is based on the administration of exogenous (mostly recombinant) enzymes into the systemic circulation to correct the biochemical defects associated with endogenous enzyme deficiencies [27]. It represents the most extended treatment strategy for LSDs, with about 20 clinical products for treatment of Gaucher disease, Fabry disease, Pompe disease, mucopolysaccharidosis of several types (I, II, IVA, VI, and VII), etc. [27]. However, current ERT suffers from the problems described above for protein therapeutics, mainly poor penetration of the administered enzymes across biological barriers in the body, limiting access to key targets such as the central nervous system [28].

Given this, drug delivery approaches have great potential to improve the therapeutic efficacy of lysosomal ERTs [27,28]. Our group was the first to investigate the use of targeted polymeric nanoparticles for enzyme delivery in LSDs [29,30,31,32,33,34]. In particular, we have focused our efforts on targeting ERTs to intercellular adhesion molecule-1 (ICAM-1), a transmembrane glycoprotein present on the endothelium and other cell types, whose expression is upregulated in many pathological states [35]. ICAM-1 targeting with polymeric NPs induces cell adhesion molecule (CAM)-mediated endocytosis, resulting in both transcytosis across the vascular endothelium and lysosomal trafficking [36,37,38]. Anti-ICAM NPs bearing acid sphingomyelinase (ASM), α-galactosidase (α-Gal), or α-glucosidase, the enzymes deficient in types A and B Niemann–Pick disease, Fabry disease, and Pompe disease, respectively, have shown enhanced enzyme delivery using various cellular models of the blood–brain barrier, neural cells, skeletal muscle cells, and endothelial cells, as well as both to brain and peripheral organs in vivo [29,30,31,32,33,34,35,36,37,38]. Potential for oral delivery was also reported using anti-ICAM NPs encapsulated in microcapsules for gastric protection and intestinal release [39,40].

Other research groups have focused on lysosomal ERT using polymeric nanoparticles. These studies include albumin–silk NPs for enhanced delivery and intracellular stability of α-Gal [41], polyelectrolyte complexes of trimethyl chitosan and α-Gal [42], particles containing guanidinylated glycosides to be able to interact with cell-surface heparan sulfate proteoglycans and deliver β-D-glucuronidase and α-L-iduronidase [43], poly(butyl-cyanoacrylate) NPs for delivery of arylsufatase B [44], etc. Among polymeric NPs, PLGA formulations have been favored by our group and others [30,34,45,46,47,48] because their acidic nature provides an additional advantage by helping to normalize the altered lysosomal pH in LSDs, shown in cells and in vivo [49].

Interestingly, enzyme loading by both surface coating and encapsulation have been explored with regard to lysosomal ERTs mediated by polymeric NPs, both showing beneficial results such as enhanced delivery to target organs in the body, increased uptake by cells, and enhanced recovery of lysosomal function [34,45,46,47,48]. However, NPs prepared by both loading methods have never been compared regarding their relative performance for this application. Each loading approach has its own advantages and disadvantages. For instance, until their release, encapsulated enzymes could be protected from the biological environment when entrapped in the polymeric matrix and prevented from reaching their substrate prior to their lysosomal release, thus avoiding premature activity, and relatively higher loading could be also expected for the entrapment method. Alternatively, surface coating by the adsorption method, for example, could protect the enzyme from being subjected to aggressive preparatory conditions of NP preparation, such as chemical solvents, sonication, or homogenization forces that may reduce their activity. On the NP surface, enzymes could readily access their substrates upon reaching the lysosome without the need for release. Since lysosomal enzymes are active at acidic but not neutral pHs, this intrinsic property would prevent premature enzyme activity prior to reaching its destination. Therefore, it is difficult to predict which loading method is more advantageous.

In this study we examined this question using the example of ICAM-1-targeted PLGA NPs, loaded with hyaluronidase (HAse) or ASM as model lysosomal enzymes.

## 2. Results

### 2.1. Characterization and Optimization of PLGA NPs Encapsulating a Model Enzyme

Our previous studies on NP-mediated ERT had used enzymes coated on the NP surface [28,29,30,31,32,33,35,37]. PLGA formulations had been preferred given their role in restoring the lysosomal pH in LSD cells [30,34,45,49]. Therefore, we sought to develop enzyme-encapsulating PLGA NPs. As a model enzyme, we used hyaluronidase (HAse), whose deficiency renders mucopolysaccharidosis type IX (OMIM 601492) [50], as its molecular weight (MW = 60 kDa), isoelectric point (4.9), and activity peak at pH 4.5–6.0 [51] are similar to many other lysosomal enzymes used for ERT [30,31,32].

First, using polyvinylalcohol (PVA) as a surfactant, we compared three different PLGA copolymers, all at 50:50 lactic acid-to-glycolic acid ratio as in our previous studies [34,45]. Copolymers included Resomer (acid terminated; 31 kDa average MW; 0.38 dL/g average viscosity), Lactel I (ester terminated; 45 kDa average MW; 0.65 dL/g average viscosity) and Lactel II (ester terminated; 68 kDa average MW; 0.85 dL/g average viscosity). The resulting NPs had sizes and encapsulation efficiency (EE, measured by the indirect method; see Materials and Methods) within a reasonable range for the intended purpose: 158-189 nm average diameter and 43–67% EE (Table 1). However, Resomer and Lactel I rendered more polydisperse formulations than Lactel II (0.5, 0.4, and 0.3 polydispersity index or PDI, respectively); hence, Lactel II was preferred.

Next, using the Lactel II copolymer, we compared three surfactants, including PVA, DMAB, and Pluronic F68. The PLGA NPs’ size averaged between 100 and 211 nm diameter, adequate for the intended application. The PVA formulation had the highest EE (59%), but it was the most polydisperse (0.3 PDI), and its neutral ζ potential (0.03 mV) was a risk factor for further aggregation (Table 1), because of which it was not preferred. Both the DMAB and the Pluronic F68 formulations had similar polydispersity (0.2 PDI) and ζ potential far from the neutral range (64 and −19 mV, respectively). Yet, the F68 formulation was preferred because of its higher EE (49% vs. 26% for the DMAB formulation) and also since NPs with positive ζ potential, such as the DMAB formulation, tend to associate non-specifically with the naturally negatively-charged elements of the cell membrane [52]. The F68 formulation could also be lyophilized in the presence of 7.5% trehalose, which would be useful for storage, and then reconstituted with only a 17% increase in hydrodynamic diameter and without appreciable aggregation (Supplementary Figure S1).

Thereafter, we optimized enzyme encapsulation for the selected Lactel II–F68 formulation (Table 2). For this purpose, we independently varied three parameters, i.e., HAse input (3.6 to 177.8 µg per mg of copolymer), co-incorporation of BSA (from 1.25:1 to 2:1 HAse to BSA) since this protein can improve drug delivery [53], and total protein input (3.6 to 267 µg per mg of copolymer). All formulations had an acceptable average diameter between 148 and 227 nm, PDI ≤ 0.2 (except formulation *b’*), and negative ζ potential (between −28 and −36 mV). We measured the EE using the indirect method employed above, based on the estimation of non-encapsulated enzyme in the supernatant after pelleting NPs (see Materials and Methods). Per the indirect method, lowering HAse input below 17.8 µg per mg of copolymer almost doubled the EE of the formulations (from ≤ 58.7 to ≥ 86.2). Surprisingly, when the EE was measured by the direct method, this trend was not as clear and encapsulation was much lower than observed by the indirect method (all formulations had ≤ 29.4% EE).

We trusted this direct method to be more reliable and conservative and, hence, used it to examine the role of varying protein input. Adding BSA during NP preparation improved HAse % EE significantly (42% increase comparing formulations *a* vs. *b* and 24% for *a*′ vs. *b*′ in Table 2). Yet, enzyme loading correlated more with total HAse input than total protein input (compare formulations *b* vs. *c*). In fact, increasing the original BSA-to-HAse ratio by 1.5-fold without changing HAse input did not affect % EE (compare formulations *c* vs. *d*). Yet, the total protein input also had an influence when large amounts of protein were used (compare *e* vs. *f* vs. *a)*.

All following experiments, which were conducted concomitantly to this optimization, used formulations *a* or *e* (Table 2). Importantly, these NPs were similar (not statistically different) in diameter, PDI, ζ potential, and enzyme loading. Thus, they can be considered as the same formulation from the perspective of NP output, where only protein input varied.

### 2.2. Activity, Antibody Coating, and Stability of the PLGA NPs Encapsulating a Model Enzyme

Next, we characterized other relevant parameters, such as the enzymatic activity, the ability to surface-coat targeting antibodies on these NPs, and the stability of these formulations in conditions mimicking storage vs. physiological conditions.

**Figure 1:**
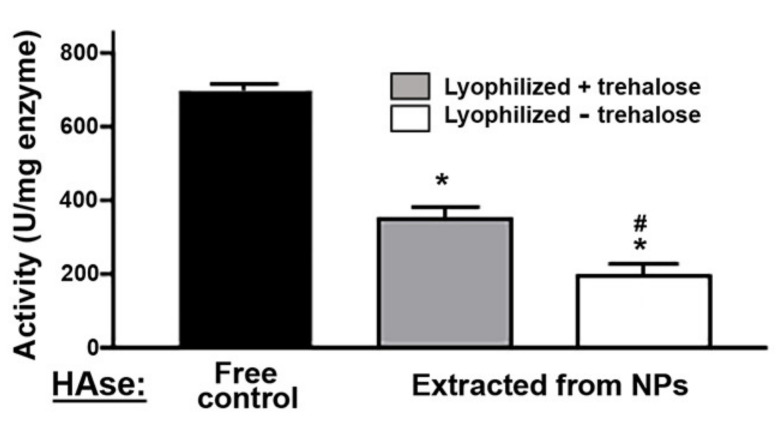
Enzyme activity of hyaluronidase (HAse) encapsulated in poly(lactide-co-glycolide) (PLGA) nanoparticles (NPs). HAse encapsulated in Lactel II–F68 PLGA NPs was extracted from NPs after lyophilization in the presence vs. absence of 7.5% trehalose. Activity was assessed by incubation for 10 min at 37 °C and pH 4.2 with a solution containing 0.4 mg/mL hyaluronic acid (HA), after which the HA content left undegraded was measured by absorbance at 540 nm. The activity of free, non-encapsulated HAse is shown as a control. Data are the mean ± standard error of the mean (SEM) (*n* ≥ 3). * Compared to free HAse control; # Compared to trehalose (Student’s *t*-test; *p* < 0.05).

With regard to enzymatic activity, this was based on the measurement of the reduction in optical density caused by HAse to a solution containing hyaluronic acid (HA; Supplementary Figure S2), as the enzyme degrades this substrate. The whole process of enzyme encapsulation in PLGA NPs, lyophilization to facilitate storage, and enzyme extraction from NPs upon dissolving them (see Materials and Methods) reduced HAse’s specific activity compared to control free enzyme (Figure 1). When NPs were lyophilized in the absence of cryoprotectants, the specific activity of the extracted enzyme decayed by 72%, while in the presence of 7.5% trehalose, this parameter decayed by 50%, showing a greater protection of the enzyme cargo under this condition.

Coating with targeting antibodies (Table 3) was performed by surface adsorption, as in our prior studies, using PLGA NPs for lysosomal ERT [30,34,45] to keep this parameter constant. While a clinical formulation would likely require antibody conjugation, the high reproducibility, stability, and targeting demonstrated in cell cultures and animal models support the use of this method as a proof-of-concept [29,30,31,32,33,34,35,36,37,38,39,40]. Coating with antibodies targeted to either human ICAM-1 for cellular experiments or mouse ICAM-1 for in vivo analysis was efficient for the preferred Lactel II–F68 formulations but not Lactel II–PVA NPs: the latter formulation had only 3.4% and 6.4% coating levels compared to the former one, for respective antibodies (after mechanical stress test). Significant antibody levels remained on the coat of the Lactel II–F68 formulations upon incubation in 50% serum to mimic physiological conditions, with a final coat of 122 and 253 antibody molecules per NP for formulations aimed to target human or mouse ICAM-1, respectively (Table 3).

**Figure 2:**
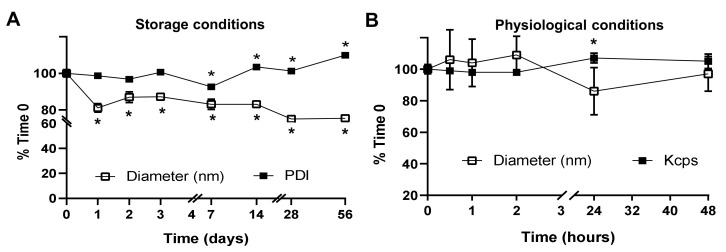
Stability of anti-ICAM PLGA NPs encapsulating HAse. Lactel II–F68 PLGA NPs encapsulating HAse and coated with anti-ICAM were incubated for the indicated time periods (**A**) at 4 °C in phosphate buffer saline (PBS) to mimic storage conditions or (**B**) at 37 °C in 50% fetal bovine serum to mimic physiological conditions. Samples were analyzed by dynamic light scattering (DLS) to determine their average hydrodynamic diameter, polydispersity index (PDI), and associated kilo counts per second (kcps) compared to the original formulation (Time 0). Data are the mean ± SEM (non-visible error bars are hidden by the data symbol). Statistics were assessed by Student’s *t*-test (*p* < 0.05). * Compares each time point to Time 0 control.

Additionally, the stability of the preferred Lactel II–F68 formulation was tested. This was first assessed at storage conditions represented by incubation at 4 °C in the suspension buffer, PBS (Figure 2A). The data demonstrated acceptable NP stability with only about a 20% decrease in the average hydrodynamic diameter up to eight weeks of storage, the latest time point tested, and a 10% to 15% increase in PDI after the first week. We next evaluated NP stability under physiological conditions represented by incubation at 37 °C in 50% serum (Figure 2B). In this case, we followed stability for 48 h because our prior in vivo results using PLGA NPs had demonstrated maximum targeting and blood clearance much earlier, between 30 and 60 min [30,34,45]. Results demonstrated great NP stability within this time frame (Figure 2B), with no apparent changes in size. As for the PDI, we were unable to reliably measure this parameter in the presence of serum, for which we alternatively focused on DLS kilo counts per second (kcps), a parameter that depends on NP size and concentration in suspension. Since our data indicated no changes in NP size, this parameter would then depend solely on the NP concentration in suspension. We observed no changes in kcps values, ruling out aggregation and precipitation of NPs incubated under physiological conditions.

### 2.3. Cellular Interactions and Intracellular Enzyme Delivery by anti-ICAM PLGA NPs

**Figure 3:**
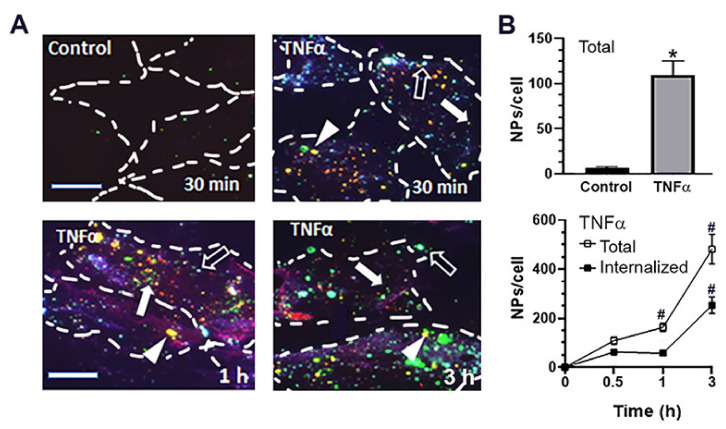
Cellular internalization of PLGA NPs encapsulating HAse. (**A**) Fluorescence microcopy pictures and (**B**) quantification of control vs. tumor necrosis factor α (TNFα)-activated human umbilical vein endothelial cells (HUVECs) incubated for the indicated time points with Lactel II–F68 NPs encapsulating AlexaFluor488-HAse (green) and coated with anti-ICAM. Cells were washed to remove non-bound NPs, fixed, and cell-surface NPs were stained using goat anti-mouse IgG labeled with AlexaFluor350 (blue). Cells were then permeabilized and all NPs were stained with goat anti-mouse IgG labeled with Texas Red (red). (**A**) Cell-surface NPs with anti-ICAM coat appear green + blue + red = white (open arrow), internalized NPs with anti-ICAM coat appear green + red = yellow (arrowheads), and green NPs are internalized and lack anti-ICAM coat, likely degraded in lysosomes (arrows). Dashed lines = cell borders. Scale bar = 10 μm. (**B**) Quantification of total and internalized NPs. Data are the mean ± SEM (non-visible error bars are hidden by the data symbol). Statistics were assessed by Student’s *t*-test (*p* < 0.05). * Compared to control; # Compared to the previous time point.

We then examined the cellular interactions of Lactel II–F68 NPs encapsulating HAse and coated with anti-ICAM, for which we used endothelial cells, as in our prior studies [29,31,32,33,45]. First, using fluorescence microscopy analysis of formulations where HAse had been labeled with green AlexaFluor488 (211 nm diameter and 0.28 PDI), we observed that NPs preferentially associated with tumor necrosis factor α (TNFα)-treated cells vs. control cells (118 vs. 6 NPs/cell at 30 min; Figure 3A,B, top panel). This was expected because ICAM-1 expression is low in basal conditions but high in diseases involving inflammation [35], mimicked by TNFα. In fact, flow cytometry of these cells validated their low vs. high ICAM-1 expression levels (not shown). Additionally, green HAse clearly colocalized with anti-ICAM on the coat of NPs located at the cell surface, which were stained in blue and red (green + blue + red = white NPs marked by open arrows in Figure 3A), further verifying that NPs carried both components. With time (Figure 3A,B, bottom panel), both total cell association and internalization increased so that, by 3 h, we found 537 total and 254 internalized NPs per cell (contrary to cell-surface NPs, internalized NPs lack blue staining, so they can be distinguished). Importantly, internalized HAse marked in green also showed colocalization with anti-ICAM marked in red (green + red = yellow NPs marked by white arrowheads in Figure 3A), e.g., at 1 h, 80% of all HAse-positive NPs were also positive for anti-ICAM.

**Figure 4:**
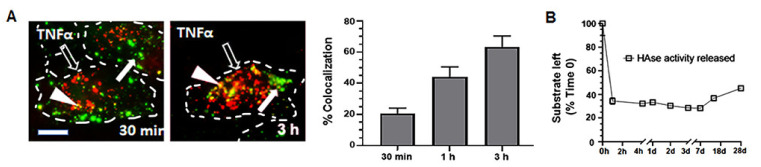
Lysosomal trafficking and released activity of PLGA NPs encapsulating HAse. (**A**) Fluorescence microcopy pictures (left) and quantification (right) of TNFα-activated HUVECs incubated for the indicated time points with Lactel II–F68 PLGA NPs coated with anti-ICAM and encapsulating AlexaFluor488-HAse (green; white arrows). Cell lysosomes had been labeled with TexasRed dextran (red; open arrows) prior to NP incubation, so that colocalization of HAse with dextran-labeled lysosomes would appear as green + red = yellow color (white arrowheads). Dashed lines = cell borders. Scale bar = 10 μm. (**B**) Quantification of HA-degrading HAse activity released from NPs upon in vitro incubation for the indicated time points under conditions mimicking the lysosomal environment (lysosomal-simulating fluid, 37 °C and pH 4.5). Enzyme activity was measured as in Figure 1. Data are the mean ± SEM.

Interestingly, we observed that the number of HAse-positive NPs colocalizing with anti-ICAM decreased around 25% between 1 h and 3 h (not shown). Since our previous studies have demonstrated that ICAM-1-targeted NPs reach lysosomes by this time, where anti-ICAM is degraded [54], this result suggested lysosomal trafficking of this formulation, its intended destination. We verified this by incubating cells with anti-ICAM PLGA NPs encapsulating green HAse and colocalizing them with red-marked lysosomes (Figure 4A). As expected, lysosomal colocalization, observed in yellow by fluorescence microscopy, increased with time and reached 63.3% by 3 h.

Additionally, we incubated NPs in test tubes at lysosomal mimicking conditions (see Materials and Methods) and measured HAse activity released from NPs over time. We found bulk release of HAse activity as fast as 1 h after incubation in lysosomal-simulating fluid at 37 °C and pH 4.5, which degraded 66–72% HA present in the reaction (28–34% HA left undegraded; Figure 4B) between 1 h and 1 week. Afterwards, the HA degradation capacity of HAse released from NPs slowly decreased from 72% to 45% between 1 week and 4 weeks (HA left undegraded increased from 28% to 55% in independent reactions; Figure 4B). Therefore, this formulation is capable of fast release of active HAse, which remains active for a significant period of time, under lysosomal conditions.

### 2.4. Comparative Characterization and Enzyme Delivery in Cells and Mice of Enzyme-Encapsulating vs. Enzyme-Coating Anti-ICAM PLGA NPs

Since all prior experiments indicated that anti-ICAM PLGA NPs encapsulating enzyme could be successfully prepared and behaved similarly to our previous enzyme-coated NPs [29,31,32,33,45], we finally compared both types of formulations side by side. We first prepared pristine Lactel II–F68 PLGA NPs (Table 4), which were smaller than the previous HAse-encapsulating formulation (111 vs. 181 nm diameter), as expected because of the absence of cargo; yet, the formulation had a similar PDI (0.16 vs. 0.15) and ζ potential (−34 vs. −36 mV). The lack of change in ζ potential suggested that enzyme cargo was not located on the surface of encapsulating NPs, which was further validated by the fact that Fourier-transform infrared (FTIR) spectroscopy showed similar spectra for both pristine and HAse-encapsulating NPs (Supplementary Figure S3). Yet, enzyme presence was measured using ^125^I-labeled HAse for a reliable quantification, rendering 538 HAse molecules/NP. Then, when pristine NPs were coated with HAse cargo and targeting anti-ICAM, their diameter increased as expected (from 111 to 141 nm), although their PDI remained similarly low (0.15). This formulation contained 292 HAse molecules/NPs, lower than encapsulating NPs, and its ζ potential changed from −34 to −26 mV, indicating surface coating. Similarly, coating HAse-encapsulating NPs with anti-ICAM increased their diameter as expected (from 181 to 236 nm), their PDI increased but remained low (0.2), and their ζ potential changed from −36 to −29 mV (Table 4), indicating surface coating. In both cases, the FTIR spectra of coated formulations were similarly altered compared to respective non-coated formulations (Supplementary Figure S3).

**Figure 5:**
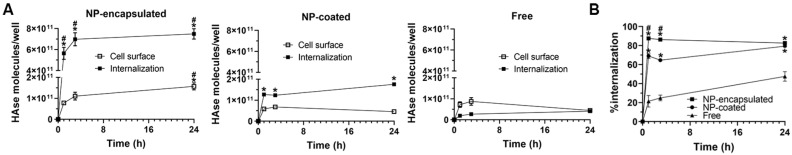
HAse delivery to cells by encapsulated vs. coated anti-ICAM PLGA NP formulations. TNFα-treated HUVECs were incubated with anti-ICAM PLGA NPs bearing 2.25 µg/mL encapsulated vs. coated ^125^I-HAse, compared to the same dose of free ^125^I-HAse. (**A**) ^125^I-HAse delivered to cells was quantified before and after removal of the cell-surface fraction by acid glycine solution to determine internalized ^125^I-HAse. (**B**) Enzyme internalization expressed as percentage of the total (surface + internal) ^125^I-HAse associated to cells. Data are the mean ± SEM (*n* ≥ 4; non-visible error bars are hidden by the data symbol) and statistics were assessed by Student’s *t*-test (*p* < 0.05). * Compares each NP formulation vs. free ^125^I-HAse at respective times and locations; # Compares NP encapsulated vs. coated enzyme for respective times and locations.

After NP characterization, formulations were tested in biological models. First, anti-ICAM NPs carrying either encapsulated or coated HAse were incubated with TNFα-activated endothelial cells to determine their respective enzyme deliveries over time and compare them to control free HAse (^125^I-HAse was traced in all three cases, whose dose was kept constant). A greater amount of HAse was found on the cell surface and internalized when the enzyme was provided as an NP formulation, with encapsulated preparations surpassing coated ones (Figure 5A): by 24 h, 3.2-fold and 18.4-fold increases were seen for surface and internalized HAse for encapsulating formulations vs. free enzyme, while a 4.3-fold increase regarding internalized HAse (no change for cell-surface counterpart) was detected for coated formulations vs. free enzyme. The rate of intracellular enzyme uptake, expressed as a percentage of the total HAse associated to cells, was also higher for NP-encapsulated vs. coated enzyme and both NP formulations surpassed free HAse (Figure 5B): respective uptake was 83%, 79%, and 47% by 24 h.

Finally, we used the same two types of formulations but substituted the HAse enzyme with recombinant acid sphingomylinase (ASM), on which a current clinical trial for enzyme replacement therapy is based (https://clinicaltrials.gov/ct2/show/NCT02004691; access on 20 February 2022). The endogenous ASM enzyme is genetically altered in an LSD called ASM deficiency (OMIM 257200 and 607616) [55]. This disease associates with clinical phenotypes historically known as types A or neurological and B or visceral Niemann–Pick disease (NPD), respectively affecting the brain or peripheral organs such as the lungs, liver and spleen [55]. Yet, patients’ symptoms commonly evolve within the continuum spectrum of manifestations between these two extremes, for which targeting as many organs as possible would be beneficial [55]. These pathological abnormalities are well reflected in the ASM knockout (ASMKO) mouse, for which this is an excellent animal model used to study therapeutic applications aimed to treat NPD [56]. Importantly, our previous publications have shown that targeting ASM to ICAM-1 by enzyme coating on the surface of model polystyrene and PLGA NPs enhanced its delivery in cell cultures, as well as in the brain and lungs in the ASMKO and wildtype mouse models [29,30,33,34,38].

Therefore, we formulated anti-ICAM PLGA (Lactel II–F68) NPs encapsulating vs. coating recombinant ASM similarly to the HAse formulations described above. Coated NPs had 231 nm average diameters, 0.24 PDI, and 51 ASM molecules/NP (Supplementary Table S1). Comparatively, encapsulating formulations had 353 nm average diameters, 0.20 PDI, and 308 ASM molecules/NP (Supplementary Table S1).

First, as a control to verify whether coated preparations behaved as in our previous studies [30,34], we injected NPs coated with ^125^I-ASM intravenously (i.v.) in wildtype vs. ASMKO mice and determined the specificity index (fold increase) over free ^125^I-ASM control (Table 5). The specificity index reflects the biodistribution gain of NP–enzyme over free enzyme, taking into account (i) the weight of the organ to which it refers, so that it reflects an organ concentration, and (ii) the circulating formulation, so that tissue-over-blood is reflected (see Materials and Methods). We are not displaying other parameters as they were similar to those characterized in our previous publications using the same formulation [30,34], and this test was meant as a verifying control, not a duplication of previously described studies. Specificity index results demonstrated that NPs increased ASM delivery to all organs (Table 5), particularly the lungs, where this formulation surpassed free ASM by 34-fold in ASMKO mice, followed by the spleen (4.6-fold), kidney (3.2-fold), liver (2.9-fold), brain (2.9-fold), and heart (2.2-fold). Because most of these organs, particularly the lungs, brain, liver, and spleen, represent main NPD targets, this result indicates a clear advantage of administering ASM in an NP preparation.

**Figure 6:**
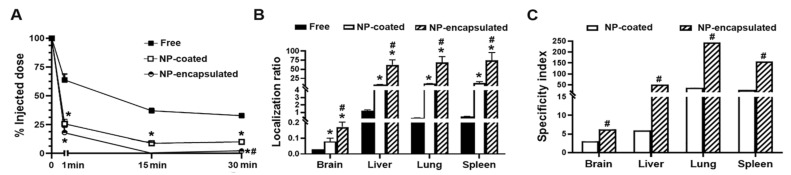
In vivo biodistribution of ASM encapsulated vs. coated anti-ICAM PLGA NP formulations. Wildtype C57B/6 mice were i.v.-injected with 200 µg/kg body weight ^125^I-ASM, administered either as a free enzyme or coated on or encapsulated in anti-ICAM PLGA NPs. (**A**) Enzyme presence in blood was analyzed at the indicated times using a gamma counter and expressed as the percentage of the injected dose (% ID). (**B**) Enzyme biodistribution in the indicated organs was assessed 30 min after injection and expressed as the tissue-to-blood localization ratio (see Materials and Methods). (**C**) The specificity index was calculated as the localization ratio of respective NP formulation over the localization ratio of the free enzyme. Data are the mean ± SEM (*n* ≥ 4; non-visible error bars are hidden by the data symbol) and statistics were assessed by Student’s *t*-test (*p* < 0.05). * Compares each NP formulation vs. free ^125^I-ASM at respective times and locations; # Compares NP encapsulated vs. coated enzyme for respective times and locations.

Data from wildtype mice were similar to those obtained in ASMKO mice (Table 5). This was somewhat expected because spread multi-system disease in the ASMKO model may increase ICAM-1 expression broadly in the body [29,55], resulting in a similar relative distribution (the represented parameter is calculated from the percentage of the injected dose (ID), so a proportional enhancement in most organs would not be noticed). This result suggests that both animal models are suitable to study the gain in biodistribution by NP formulations. Given this and considering the higher cost and difficulty in obtaining ASMKO mice, we used wildtype mice to compare NP formulations bearing ASM on the coat to those where ASM was encapsulated (Figure 6). We found higher circulating levels for free enzyme, while both NP preparations were cleared from the circulation within the first 30 min: 10 and 2.1% ID for coated and encapsulated formulations, respectively; 3.2- and 15.5- fold below free enzyme levels (Figure 6A). We have observed this phenomenon for all ICAM-1 targeted formulations we have tested and have verified it to be due to fast ICAM-1 targeting, since its expression on the vascular endothelium of all organs makes it readily accessible for binding [30,31,32,33,38]. In fact, by this time, 59 and 83% ID was cumulatively found in all organs for the coated and encapsulated formulations, while only 28% ID was found for free enzyme (not shown), even though this species was higher in circulation (Figure 6A). Biodistribution calculated as % ID per gram of organ, indicative of organ concentration, showed the lungs to be the main target for ASM administered as NP formulations: 60 and 58% ID/g for coated and encapsulated preparations, respectively; 10- and 9.8-fold increases compared to free ASM (Supplementary Table S2). This organ was followed by the spleen (54 and 61% ID/g, respectively, 5.6- and 6.3-fold increase over free ASM) and the liver (36 and 51% ID/g, respectively, 1.4- and 2-fold increase over free ASM).

Additionally, we calculated the localization ratio (see Materials and Methods), which normalizes the biodistribution in an organ to the circulating fraction and, thus, renders the information more reliable on the tissue-targeting capacity of formulations with different circulation rates (Figure 6B and Supplementary Table S3). This parameter clearly showed improved delivery of ASM carried by NP formulations, with encapsulated preparations surpassing coated ones: lung localization ratios were 9.9 for coated ASM, 68.2 for encapsulated ASM, and 0.28 for free ASM. The specificity index (localization ratio or the NP–enzyme over free enzyme; Figure 6C and Supplementary Table S4) indicated that coated formulations enhanced ASM biodistribution in all organs except the heart, indicating increases of 35.6-fold in the lungs and 3.0-fold in the brain, two of the main targets in NPD. Encapsulated formulations showed further improved results, e.g., 244-fold increase in the lungs and a 6.2-fold increase in the brain.

**Figure 7:**
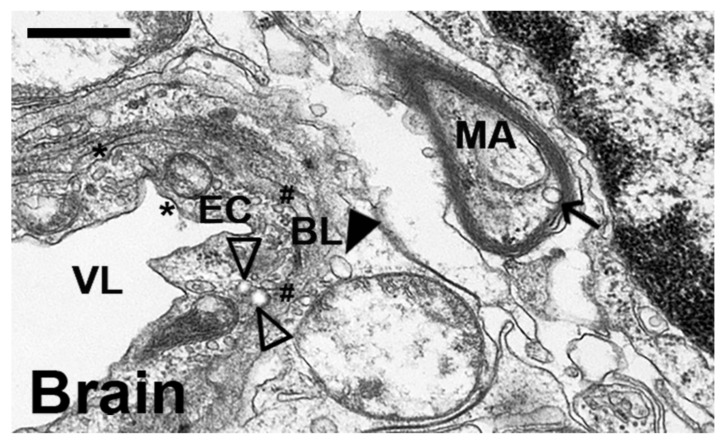
Visualization of coated anti-ICAM/ASM PLGA NPs in mouse brain. Wildtype C57B/6 mice were i.v.-injected similarly to those in Figure 6, followed by isolation of brain samples 3 h after NP administration and processing for transmission electron microscopy. BL = basal lamina; EC = endothelial cell; MA = myelinated axon; VL = (blood) vessel lumen. Open arrowheads = NPs close to the abluminal side of an endothelial cell; closed arrowheads = NP located passed the endothelium and basal lamina; arrow = NP within the myelinated axon of a neuron. * = clathrin-coated pits. # = caveolae-like vesicles. Scale bar = 500 nm.

Finally, transmission electron microscopy of postmortem samples verified the presence of NPs in target tissue (Figure 7). In particular, we focused on the brain, since delivery to this organ is the main obstacle for current ERTs aimed to treat LSDs, including NPD [27,28,55]. Despite selecting coated particles to be conservative, which had enhanced brain delivery less prominently than encapsulating counterparts, NPs were clearly visible within the brain endothelium (open arrowheads), past this layer and the basal lamina (closed arrowhead), and within neurons (an example within a myelinated axon is marked by the arrow).

## 3. Discussion

ERT for LSDs is a relevant example of protein therapeutics whose pharmaceutical performance is in need of improvement [26,27,28]. PLGA NPs represent an interesting alternative for this goal [30,34,45,46,47,48]. The objective of this study was to compare, for the first time, PLGA NPs bearing enzyme cargo either encapsulated or surface-coated, as previous publications from different laboratories have demonstrated good potential for both types of formulations [30,34,45,46,47,48]. We used ICAM-1-targeted NPs that had shown promising results in cell culture and mouse models regarding enhanced enzyme delivery throughout the body as needed for this application, increased intracellular trafficking to lysosomes, and enhanced substrate degradation effects [29,30,31,32,33,34,35,36,37,38].

First, the water-in-oil-in-water (W/O/W) double emulsion technique was employed to produce stable NPs containing a hydrophilic enzyme model, HAse, as this method has been shown to achieve significant entrapment efficiencies over single emulsification procedures [57]. Three PLGA copolymers with different terminal groups, MW, and inherent viscosities were compared, since these parameters are known to influence the entrapment efficiency of therapeutics, NP size, and biodegradation profile [57,58,59]. All three copolymers resulted in a desirable NP size (≤211 nm diameter), yet Resomer associated with higher EE % (Table 1). This has been reported for other proteins [60] and could be attributed to the relatively higher hydrophilic nature of this copolymer and charge interaction between its free carboxyl end-groups and positive HAse surface [51]. However, higher PDI values observed for Resomer NPs, as well as Lactel I NPs (Table 1), favored the selection of Lactel II. The latter copolymer was used with non-ionic (PVA and F68) surfactants and a cationic (DMAB) surfactant, given their different mechanisms of action. PVA interpenetrates PLGA molecules, forming a stable coating network over its surface; F68 reduces NP surface tension by orienting at the interface of the o/w emulsion; and the DMAB’s cationic nature prevents the coalescence of NPs by electrostatic repulsion [61,62]. Though DMAB-stabilized NPs were monodispersed and ≤100 nm in diameter, poor EE % and highly positive ζ potential turned down its selection because positively charged NPs often result in non-specific interactions with naturally negatively charged cell membranes [52,63]. PVA-stabilized NPs were discarded because of their almost neutral ζ potential, which represents a higher risk for aggregation; hence, F68-stabilized NPs were preferred (Table 1).

Next, HAse loading was optimized by varying protein input relative to the copolymer mass and/or by mixing it with BSA at different ratios (Table 2). Curiously, EE results obtained from direct vs. indirect determination methods were quite different, with direct methods showing much lower values. While we cannot fully explain this phenomenon, it is possible that inefficient HAse extraction from NPs to determine protein concentration and/or incomplete NP harvest by centrifugation might contribute to this observation. Other studies have found similar discrepancies [64]. Nevertheless, this sheds light on the need to analyze these formulations using the direct method to conservatively estimate EE. Using this method, we observed that the use of BSA for NP preparation was beneficial, as in other studies where albumin has been reported to provide an advantage and to protect encapsulated enzymes from interfacial inactivation [53,65].

Using a direct method for the determination of encapsulated enzyme, we identified a maximal EE of ≈30% at 8.9 µg HAse and 4.4 µg BSA per mg of copolymer (Table 2). In comparison, other groups designing PLGA NPs for treatment of LSDs have found 45–70% EE using direct methods, particularly when encapsulating either albumin or galactosylceramidase alone [46,47]. Other groups have reported 70–75% EE for enzyme encapsulation in PLGA NPs using indirect methods for this determination [65,66], while when we used similar indirect methods, our best-performing formulation had >90% EE. At peak, our best formulations contained 538 HAse molecules/NP and 308 ASM molecules/NP. This difference may be due to the slightly higher MW of ASM (70–75 kDa vs. 60 kDa for HAse) and the fact that this enzyme is often found in dimeric and tetrameric forms [67], which may pose stronger steric hindrance during encapsulation. Importantly, encapsulation offered a clear advantage for enzyme loading compared to surface coating, as enzyme-coated NPs had 292 and 51 enzyme molecules/NP for HAse and ASM, respectively (Table S1).

Additionally, encapsulated formulations could also be successfully coated with targeting anti-ICAM (Table 3) at an extent similar to that observed for non-encapsulating PLGA NPs. For instance, in this study, we found a coating density of 443 antibody molecules/NP for HAse-encapsulating formulations. Taking into account the NP size prior to antibody coating (Table 4) and modeling an IgG molecule as a 12 nm sphere [68], one would expect 229 antibody molecules/NP for a monolayer coat, implying that NPs may bear more than one antibody layer. Incubation in serum decreased coating down to 253 antibody molecules/NP, in line with a monolayer coat. This is within the range of our previous reports, such as non-encapsulating PLGA formulations bearing 220-313 antibody molecules/NP, for example [45,69]. Polymeric NPs coated with ≈ 200 anti-ICAM antibody molecules/NP have shown significant ICAM-1 targeting, endocytosis, and transcytosis in cellular and animal models, including delivery of lysosomal enzymes such as ASM to the lungs and the brain [30,31,32,34,38]. Similarly, this level of antibody coating achieved for enzyme-encapsulated formulations provided significantly enhanced enzyme delivery both in cellular and animal models. Binding to cells in culture and organs in mice was fast, i.e., >100 NP/cell and >80% ID in 30 min, as shown in Figure 5 and Figure 6. Nevertheless, antibody coating was used here as a model, not intended for translational development. Since this aspect was similar for the formulations being compared and clear cellular and in vivo targeting was detected (discussed below), this model is still valid for the intended purpose: the comparison of enzyme-encapsulated vs. enzyme-coated NPs.

Importantly, albeit the observed 50% reduction in specific activity, enzyme encapsulation in NPs enhanced intracellular delivery by 18.4-fold, brain delivery by 6-fold and lung delivery by 244-fold (further discussed below), because of which encapsulation remains highly advantageous. One must also consider the fact that determination of the specific activity of the encapsulated enzyme required extraction from NPs, involving NP dissolution in acetonitrile, which may per se be the cause of the reduction in specific activity of the enzyme. These formulations could also be lyophilized for storage and resuspended without significantly affecting NP properties and retaining enzymatic activity, particularly in the presence of trehalose (Figure 1). The high glass transition temperature of this cryoprotectant is known to prevent irreversible aggregation of NP during freeze-drying [70], and through hydrogen bonding, this saccharide confers structural protection to proteins, preserving their activity [71,72]. In addition, NPs remained reasonably stable for two months under 4 °C storage conditions in suspension (Figure 2A). Apart from antibody shedding discussed above, which still allowed abundant targeting, other NP parameters remained stable for two days in serum (Figure 2B), a time when cell culture and in vivo targeting (Figure 5 and Figure 6) have occurred. The activity of the enzyme released from NPs under lysosomal conditions was sustained for a week and then only decayed ≈1.5-fold by 4 weeks, demonstrating the significant potential of this formulation.

Finally, cell uptake and lysosomal trafficking of anti-ICAM NPs encapsulating an enzyme were efficient and comparable to that of enzyme-coated formulations we have previously reported [29,31,33,45]. For instance, we found ≈ 80% of the enzyme NPs to be internalized by cells within 1 h (Figure 5B) and 63% to colocalize with lysosomes by 3 h (Figure 4A), which is fully consistent with the range of uptake and lysosomal colocalization reported in our previous publications where enzymes had been coated on anti-ICAM NPs [29,31,32,33]. Therefore, both encapsulated and coated formulations seem to induce similarly efficient intracellular trafficking. However, likely because of the greater enzyme loading of encapsulated formulations (308 encapsulated vs. 51 coated ASM molecules and 539 encapsulated vs. 292 coated HAse molecules; Table S1) and possibly because the presence of enzyme molecules on the NP coat may interfere with antibody binding to its cell-surface receptor, encapsulated formulations surpassed coated ones in terms of absolute intracellular enzyme delivery (Figure 5). Yet, enzyme-coated formulations also improved intracellular enzyme delivery compared to free counterparts (Figure 5). A similar trend was observed in mouse models (Figure 6B,C), where both NP formulations considerably enhanced the enzyme biodistribution to all organs, as required for a lysosomal ERT application [27,28]. In fact, NPs were visualized inside cells of target organs, e.g., both within the endothelium and beyond this cellular barrier within neurons in the brain (Figure 7), the main obstacle for current ERTs. As in cellular models, encapsulated formulations surpassed the enzyme delivery efficacy of coated ones (35- and 244-fold improvements in the lungs, respectively, and 3- vs. 6-fold improvements in the brain; Figure 6C).

## 4. Materials and Methods

### 4.1. Reagents

Resomer 503H PLGA (50:50 copolymer ratio, acid terminated, 24–38 kDa average molecular weight (MW), 0.32-0.44 dL/g viscosity) and Lactel I (50:50, ester terminated, 30–60 kDa average MW, 0.55–0.75 dL/g) were from Sigma-Aldrich (St. Louis, MI, USA). Lactel II PLGA (50:50, ester terminated, 55–80 kDa average MW, 0.76–0.94 dL/g) were purchased from Evonik (Birmingham, AL, USA). Mouse monoclonal anti-human ICAM-1 (clone R6.5) and rat monoclonal anti-mouse ICAM-1 (clone YN1) were obtained from hybridomas from American Type Culture Collection (Manassas, VA, USA) and purified at NANBIOSIS Customs Antibody Service (Barcelona, Spain). Control mouse and rat immunoglobulin G (IgG) were from Calbiochem (San Diego, CA, USA) and fluorescently-labeled secondary antibodies were from Jackson Immunoresearch (West Grove, PA, USA). ^125^I and Pierce Iodogen were from Thermo Fisher Scientific (Waltham, MA, USA), and BioSpin Tris Columns were from BioRad (Hercules, CA, USA). BSA was from Fisher Scientific (Waltham, MA, USA). Recombinant ASM was kindly provided by Dr. Edward H. Schuchman from the Department of Genetics and Genomics Sciences in Mount Sinai School of Medicine (New York, NY, USA). TexasRed dextran (10 kDa) was from Molecular Probes (Carlsbad, CA, USA). The 1000-2200 kDa HA, HAse Type I-S from bovine testes, 87–90% hydrolized PVA, DMAB, Pluronic F68, trehalose, and all other reagents were from Sigma (St. Louis, MO, USA).

### 4.2. Labeling of Antibodies and Enzymes

Where indicated, anti-ICAM (R6.5 or YN1) or enzymes (HAse or ASM) were labeled with ^125^Iodine (^125^I) or AlexaFluor 488 for radioisotopic quantification or fluorescence tracing, respectively. ^125^I was conjugated to antibody or enzymes by incubation at 1 mg/mL for 5 min at 4 °C with 20 µCi ^125^I using Pierce Iodogen. Free ^125^I was then removed using BioSpin Tris Columns (BioRad; Hercules, CA, USA). Trichloroacetic acid precipitation of the protein followed by (i) centrifugation to separate protein in the pellet vs. free ^125^I in the supernatant and (ii) determination of the label and protein concentration in the pellet, were used to determine the protein specific activity (cpms/mass).

HAse labeling with green, fluorescent AlexaFluor488 was completed by conjugation. Briefly, 1 M sodium bicarbonate was added to 0.5 mL of 2 mg/mL HAse solution to raise the pH up to 8.3. Reactive dye from the labeling kit was then added to the solution and the reaction was stirred at room temperature for 1 h, after which non-conjugated dye was removed by size exclusion chromatography following vendor’s instructions.

### 4.3. Preparation of HAse NPs

PLGA NPs encapsulating non-labeled, AlexaFluor488-labeled, or ^125^I-labeled HAse were prepared using either Resomer, Lactel I, or Lactel II copolymers (all 50:50 glycolic-to-lactic ratio) and either Pluronic F68, PVA, or DMAB surfactants. NPs encapsulating ^125^I-labeled ASM were prepared using Lactel II and Pluronic F68. In all cases, NPs were prepared using the double-emulsion solvent evaporation method. An aqueous phase containing 1.1:80 mg/mL protein (either enzyme alone or a 2:1 or 1.25:1 mixture of enzyme + BSA) in water was added dropwise under stirring to an organic phase containing 18 mg/mL copolymer dissolved in ethyl acetate. This primary emulsion was homogenized by stirring and sonication on ice and then added dropwise to an aqueous solution containing either 2% F68, 2% PVA, or 0.5% DMAB, as indicated, to form a secondary emulsion, which was also homogenized by stirring and sonication. The solvent was removed by evaporation and NPs were collected by centrifugation.

PLGA NPs coated with HAse or ASM were prepared using Lactel II and Pluronic F68. An organic phase containing 10 mg/mL copolymer dissolved in ethyl acetate was prepared, which was added dropwise to an aqueous phase containing 2% F68 surfactant, under stirring. The resulting emulsion was vortexed, sonicated, and added dropwise to an aqueous solution containing 0.2% F68 under stirring. The solvent was removed by evaporation, and NPs were collected by centrifugation, as described [30,34,45].

### 4.4. NP Characterization

The hydrodynamic size (mean diameter), PDI, ζ potential, and NP concentration of the resulting formulations were determined by DLS using a Zetasizer Ultra instrument (Malvern; Worcestershire, UK) and analyzed with associated ZS XPLORER software. Additionally, FTIR was used to examine the spectra of different formulations and infer the internal vs. surface location of enzyme cargo in respective encapsulating vs. coating NPs, using Thermo Scientific Nicolet iS^TM^ 10 FTIR spectrometer (Thermo Fisher Scientific, Waltham, MD, USA) and JCAMP data file viewer from the Polymer Science Learning Center of the University of Wisconsin (Madison, WI, USA).

### 4.5. Encapsulation Efficiency and Enzyme Extraction

Encapsulation efficiency (EE) was assessed by tracing protein concentration and the ^125^I label described above. Where indicated, the indirect or direct methods were used. For the indirect method, the NP suspension was centrifuged at 12000 rpm for 12 min to pellet down NPs, followed by quantification of free enzyme in the supernatant. The free enzyme level was then subtracted from the original enzyme input used to prepare NPs, where the difference should indicate the enzyme content in the NP pellet. For the direct method, protein was quantified after extraction from NPs. This involved lyophilization (see Section 4.6) and incubation in acetonitrile for 30 min at room temperature, followed by centrifugation at 10,000 rpm for 10 min to discard the polymer-containing supernatant. The pellet was redispersed in phosphate-buffered saline (PBS), following by centrifugation at 10,000 rpm for 10 min, after which the protein content in the PBS supernatant and the remaining pellet (dissolved in 0.1 N NaOH) were measured and added. As a verification, encapsulation of ^125^I-enzyme was directly determined in a gamma counter without NP extraction. EE was calculated as follows:

(1) $$\mathrm{EE}\;\%=\frac{\mathrm{HAse}\;\mathrm{in}\;\mathrm{the}\;\mathrm{NPs}\;\mathrm{pellet}\;\left(µg\right)}{\mathrm{HAse}\;\mathrm{added}\;\mathrm{during}\;\mathrm{NP}\;\mathrm{preparation}\;\left(µg\right)}\times 100$$

### 4.6. Lyophilization

Where indicated, NPs were lyophilized overnight at a 0.140 mbar and −45 °C in a Christ Alpha 1–4 D instrument (GmbH) without or with 7.5% trehalose. Resuspension was performed in PBS followed by sonication.

### 4.7. Enzyme Activity

HAse activity was measured for NP-encapsulated HAse after NP lyophilization and enzyme extraction (see Section 4.5 and Section 4.6) using the Worthington™ Hyaluronidase Assay [73]. This consists of tracing the reduction in optical density, at 540 nm, of a 0.4 mg/mL HA solution mixed with HAse (10 min at 37 °C and pH 5.3) and comparing this to control HA samples that did not receive the enzyme and to a standard curve of free HAse of known specific activity.

### 4.8. Coating with Targeting Antibodies or Enzyme + Antibodies

Where indicated, NPs were coated with protein, either anti-ICAM alone (against human or mouse ICAM-1) or mixed with HAse or ASM enzymes. This was achieved by mixing NPs and proteins in PBS at 0.3:1 mg/mL total protein, as in our prior studies [30,34,38,45]. At this concentration, antibodies interact with polymeric surfaces preferably through their Fc region, favoring an outward display of the variable regions [74]. After 2.5 h incubation at room temperature, non-coated protein was washed by adding PBS and NPs were harvested by centrifugation at 12,000 rpm (Eppendorf Centrifuge 5424 R), followed by resuspension in PBS containing 0.3:1% BSA and sonication to eliminate potential aggregates.

For coating quantification, either antibody or enzyme were previously labeled with ^125^I as described in Section 4.2. This allowed direct quantification of the ^125^I content in a gamma counter (PerkinElmer, Boston, MA, USA).

### 4.9. NP Stability under Storage and Physiological Conditions

Lactel II–F68 PLGA NPs encapsulating HAse and coated with anti-ICAM were incubated (i) from 1 h to 8 weeks at 4 °C in PBS and pH 7.4 to mimic storage conditions, or (ii) 30 min to 48 h at 37 °C in 50% FBS and pH 7.4 under gentle shaking, to mimic physiological conditions. At the indicated time points, samples were collected and analyzed by DLS to determine mean diameter, PDI, and kcps.

### 4.10. Cell Cultures

Human umbilical vein endothelial cells (HUVECs) pooled from different donors were cultured in M199 supplemented with 15% FBS, 2 mM L-glutamine, 15 µg/mL endothelial cell growth supplement, 100 µg/mL heparin, 100 U/mL penicillin, and 100 µg/mL streptomycin. For experiments, cells were seeded on 1% gelatin-coated glass coverslips and grown to confluence at 37 °C, 5% CO2, and 95% relative humidity. Where indicated, cells were incubated for 16 h prior to experiments with 10 ng/mL TNFα to mimic inflammation associated with many LSDs, including NPD, which is known to increase ICAM-1 expression [26,27,33,55].

### 4.11. Cellular Uptake

Control and TNFα-activated HUVECs were incubated at 37 °C from 30 min to 3 h with Lactel II–F68 PLGA NPs encapsulating AlexaFluor488-HAse (green) and coated with anti-ICAM. Cells were washed to remove non-bound NPs, fixed with 2% paraformaldehyde for 15 min, and cell-surface NPs were stained using goat anti-mouse IgG labeled with AlexaFluor350 (blue). Cells were then permeabilized with 0.1% Triton X-100 for 15 min and mouse anti-ICAM on the NP coat was detected using goat anti-mouse IgG labeled with Texas Red (red). Using this method, cell-surface NPs with anti-ICAM coat appear white when observed by fluorescence microscopy (green + blue + red), internalized NPs with anti-ICAM coatings appear yellow (green + red), and NPs that are internalized and lack anti-ICAM coat appear green alone. This can be used to quantify total number of HAse-positive particles, their cell surface vs. intracellular localization, and their respective colocalization with the NP anti-ICAM antibody coat.

Images were obtained using an Olympus IX81 microscope (Olympus, Inc., Center Valley, PA, USA), 60× oil immersion objective (UPlanApo, Olympus, Inc., Center Valley, PA, USA), ORCA-ER camera (Hamamatsu Corporation, Bridgewater, NJ, USA), and SlideBook™ 4.2 software (Intelligent Imaging Innovations, Denver, CO, USA). Each individual cell was localized using bright field and the number of NPs stained in each color was then quantified per cell from microscopy images using a custom algorithm in Image-Pro 6.3 (Media Cybernetics, Bethesda, MD, USA), as described [31,33]. Briefly, this algorithm detects all fluorescent objects that are ≥100 nm diameter (to avoid interference with molecular elements) and have fluorescent intensity clearly above the background level (upper half of all measurable range, since our NPs are typically intense). Then, the algorithm quantifies the number of NPs in each said “object” by dividing the object size (area pixels) by the number of pixels equivalent to the NP diameter [31,33].

### 4.12. Lysosomal Trafficking

TNFα-activated HUVECs were first incubated with 1 mg/mL 10 kDa TexasRed dextran (red) for 45 min at 37 °C to enable its internalization by pinocytosis. The cell medium were then removed by washing and cells were incubated for 45 min at 37 °C to allow internalized dextran to traffic to lysosomes, where it remains because of the mammalian cell inability to degrade this biopolymer. Our previous studies have confirmed full colocalization of dextran with Lamp-1 positive lysosomes by this time [29,54]. Then, cells were incubated for 30 min at 37 °C with Lactel II–F68 PLGA NPs encapsulating AlexaFluor488-HAse (green) and coated with anti-ICAM to allow NP binding to cells. Cells were washed to remove non-bound NPs from the cell medium and incubated with NP-free medium for additional time, to allow synchronized trafficking of pre-bound NPs for a total of 0.5, 1, or 3 h. Colocalization of green NPs with red lysosomes was then assessed by fluorescence microscopy using the settings described in Section 4.11, as in our prior studies [29,54].

### 4.13. Enzyme Release under Lysosomal Conditions

To study the release of HAse in lysosomal conditions, Lactel II–F68 PLGA NPs encapsulating HAse and coated with anti-ICAM were incubated under gentle shaking at 37 °C between 1 h and 4 weeks in artificial lysosomal fluid (ALF). ALF contained 0.525 mM magnesium chloride, 54.93 mM sodium chloride, 0.5 mM disodium hydrogen phosphate, 0.275 mM sodium sulfate, 0.871 mM calcium chloride dihydrate, 0.262 mM sodium citrate dihydrate, 150 mM sodium hydroxide, 108.3 mM citric acid, 0.786 glycine, 0.391 mM sodium tartrate dihydrate, 0.759 mM sodium lactate, and 0.782 mM sodium pyruvate, and its final pH was adjusted to 4.5. At the indicated time points, suspensions were centrifuged at 12,000 rpm for 15 min to separate the NP pellet, and the enzymatic activity of HAse released in the supernatant was quantified as described in Section 4.7.

### 4.14. In Vivo Biodistribution in Mice

Where indicated, C57BL/6J or ASMKO mice were anesthetized and i.v.-injected with either 200 µg/kg body weight free ^125^I-ASM or ^125^I-ASM encapsulated in or coated on anti-ICAM Lactel II–F68 PLGA NPs. At the indicated time points, blood was withdrawn from the retroorbital sinus, then mice were euthanized under anesthesia, and organs collected, weighed, and measured in a gamma counter to determine their ^125^I content. Data were used to calculate (i) percent of the injected dose (% ID), which compares as a percentage the label found in blood or an organ to the dose injected (total dose–dose remaining in the syringe); (ii) % ID per gram of organ (% ID/g), which divides the previous value per the weight of an organ to obtain a concentration-like measurement that permits comparisons among organs with very different weights; (iii) localization ratio, which is the % ID/g found in an organ divided by the % ID/g found in blood; and (iv) the specificity index, which is the localization ratio of the NP formulation divided by the localization ratio of the free enzyme [31,38,45]. These studies were performed in accordance with approved IACUC protocols and under compliance of federal, state, and University of Maryland regulations.

### 4.15. Visualization of Mouse Tissues

C57Bl/6 mice were i.v.-injected under anesthesia with anti-ICAM/ASM-coated NPs, as described above. At sacrifice (3 h), intracardial perfusion was performed, first with PBS and then a mixture of 2.5% glutaraldehyde, 4% paraformaldehyde in 0.1 M sodium cacodylate buffer to eliminate blood and fix tissues. Samples were finally processed for transmission electron microscopy, as reported [31,34].

### 4.16. Statistics

Data were calculated as mean ± standard error of the mean (SEM), where the number of repeats is indicated in respective figure legends. Statistical significance was determined as *p* < 0.05. For multiple comparisons, we used one-way ANOVA followed by Tukey’s test, while for two-group comparisons we used Student’s *t*-test, which were both run through GraphPad Prism^®^ version 8 (GraphPad Software; San Diego, CA, USA).

## 5. Conclusions

Formulating lysosomal enzymes in ICAM-targeted PLGA NPs improves their interaction with, uptake by, and lysosomal trafficking in cells, as well as their in vivo biodistribution throughout the body, including the brain and visceral organs. While said improvement is observed for both enzyme encapsulated and coated PLGA formulations, the former preparations are superior because of their enhanced enzyme load per NP and, likely, lack of interference with the targeting antibody coat. Furthermore, encapsulated formulations are reasonably stable during storage at 4 °C in suspension or lyophilized form, as well as under physiological conditions, and release active enzyme in the lysosomal environment, whose activity is sustained for several weeks. Therefore, these formulations hold considerable potential to advance lysosomal ERTs.

## Figures and Tables

**Table 1:** Role of copolymers and surfactants in nanoparticle (NP) properties.

Copolymer	Surfactant	Diameter (nm)	PDI	ζ (mV)	EE (%)
Resomer	PVA	178.1 ± 3.1	0.5 ± 0.01 *	1.6 ± 0.1	67.3 ± 3.0
Lactel I	PVA	158.3 ± 5.8 *	0.4 ± 0.03 *	1.9 ± 0.03	43.0 ± 3.6 *
Lactel II	PVA	189.1 ± 2.1 #	0.3 ± 0.01 *,#	0.03 ± 0.01 *,#	59.6 ± 2.5
Lactel II	DMAB	99.7 ± 1.1 #	0.2 ± 0.01	63.7 ± 0.6 #	26.0 ± 2.0 #
Lactel II	Pluronic F68	211.0 ± 11.0 #	0.2 ± 0.01 ns	−18.7 ± 0.2 #	49.2 ± 2.3

ζ = zeta potential. DMAB = didodecyldimethylammonium bromide. EE = encapsulation efficiency (indirect method; see Materials and Methods). PDI = polydispersity index. PVA = polyvinylalcohol. Data are the mean ± standard error of the mean (SEM) (*n* = 3). Statistics were assessed by one-way ANOVA and Tukey post-hoc test (*p* < 0.05). * Differences among the three PVA formulations; # Differences among the three Lactel II formulations. ns = non-significant difference vs. DMAB formulation.

**Table 2:** Enzyme loading optimization.

Protein Input (µg/mg Copolymer)			EE (%) (Indirect)	EE (%) (Direct)	HAse (molec/NP)	Diameter (nm)	PDI	ζ-Potential (mV)
HAse	BSA	Total						
177.8 *e*	88.9	266.7	52.1 ± 0.4	2.5 ± 0.2 $	719.0 ± 114.2	211.0 ± 11.0	0.1 ± 0.01	−30.0 ± 1.3
22.2 *f*	11.1	33.3	45.8 ± 0.9	11.5 ± 1.0 $	885.2 ± 270.8	189.3 ± 1.8	0.2 ± 0.02	ND
17.8	-	17.8	58.7 ± 1.5	14.2 ± 2.2	284.3 ± 46.7	148.0 ± 3.5	0.1 ± 0.01	−34.03 ± 1.1
8.9 *a*	4.4	13.3	92.1 ± 0.1	29.4 ± 2.4 $	538.0 ± 68.0	180.6 ± 7.9	0.1 ± 0.01	−36.57 ± 0.5
8.9 *b*	-	8.9	94.5 ± 0.1	20.7 ± 2.0 *	697.0 ± 363.0	194.1 ± 11.2	0.1 ± 0.02	−27.98 ± 0.2
7.1	3.6	10.7	86.2 ± 3.1	8.1 ± 0.8	208.5 ± 25.4	174.3 ± 2.1	0.1 ± 0.01	−35.78 ± 0.6
5.9 *c*	3	8.9	89.1 ± 0.2	12.0 ± 0.8 #	313.0 ± 165.0	172.8 ± 8.8	0.1 ± 0.01	−35.77 ± 0.3
5.9 *d*	4.7	10.7	88.0 ± 4.5	9.9 ± 2.4 ns	228.0 ± 44.0	177.3 ± 3.2	0.1 ± 0.01	−35.07 ± 0.4
3.6 *a*′	1.8	5.3	95.0 ± 0.0	14.3 ± 1.0	120.5 ± 10.5	226.8 ± 26.7	0.1 ± 0.02	−36.15 ± 0.2
3.6 *b*′	-	3.6	89.4 ± 1.1	11.5 ± 0.1 *	130.5 ± 19.5	179.0 ± 12.9	0.5 ± 0.04	−35.38 ± 0.4

BSA = bovine serum albumin. HAse = hyaluronidase. Molec = molecules. Data are the mean ± SEM (*n* ≥ 3). Statistics were assessed by Student’s *t*-test for dual comparisons or one-way ANOVA and Tukey post-hoc test when comparing more formulations (*p* < 0.05). Direct EE (%) was compared as follows: * Formulations containing the same hyaluronidase (HAse) input with or without BSA (*a* vs. *b* and *a*′ vs. *b*′); # Formulations containing BSA or not and receiving the same total protein input (8.9 µg/mg copolymer; *b* vs. *c*); ns (non-significant difference) Formulations containing the same HAse input and different BSA inputs (*c* vs. *d*); $ Formulations containing decreasing total protein input while keeping the HAse-to-BSA ratio constant (*e* vs. *f* vs. *a*).

**Table 3:** Coating of HAse-encapsulated PLGA NPs with targeting antibodies.

Nanoparticles	Ab	Original Coating		After Mechanical Stress		After Serum	
		Ab/NP	Ab/µm^2^	Ab/NP	Ab/µm^2^	Ab/NP	Ab/µm^2^
Lactel II-PVA	R65	100.3 ± 4.0 *	675.6 ± 27.0 *	23.2 ± 2.4 *	156.4 ± 16.0 *	9.5 ± 0.1 *	63.9 ± 0.9 *
Lactel II-F68	R65	1229.6 ± 29.2	7974.4 ± 189.1	679.8 ± 21.1	4408.4 ± 136.5	121.9 ± 0.0	2530.0 ± 1739.7
Lactel II-PVA	YN1	96.9 ± 7.9 #	652.9 ± 53.3 #	28.3 ± 2.4 #	190.7 ± 16.3 #	10.5 ± 0.1 #	71.0 ± 1.0 #
Lactel II-F68	YN1	880.0 ± 8.6	5706.9 ± 78.6	443.6 ± 67.5	2876.6 ± 437.7	252.9 ± 39.5	1640.1 ± 256.3

Ab = antibody. R6.5 recognizes human ICAM-1. YN1 recognizes mouse ICAM-1. Data are the mean ± SEM, where statistics were assessed by Student’s *t*-test (*p* < 0.05). * Compares PVA to respective F68 formulation for R6.5 antibody. # Compares PVA to respective F68 formulation for YN1 antibody.

**Table 4:** Characterization of PLGA NPs with vs. without enzyme cargo and targeting antibody.

PLGA Formulation	Diameter (nm)	PDI	ζ (mV)	HAse (molec./NP)
Pristine	111.3 ± 3.7	0.16 ± 0.06	−33.7 ± 1.9	N/A
HAse and anti-ICAM coat	140.7 ± 10.9	0.15 ± 0.02	−25.6 ± 5.9	291.6 ± 54.1
Encapsulated HAse	180.6 ± 7.9	0.15 ± 0.01	−36.5 ± 0.5	538.0 ± 68.0
Encapsulated HAse and anti-ICAM coat	236.1 ± 30.2	0.21 ± 0.02	−29.8 ± 6.7	(as above)

N/A = not applicable. All formulations used PLGA Lactel II co-polymer and F68 surfactant. ζ = zeta potential. Data are the mean ± SEM (*n* = 3).

**Table 5:** Specificity index of NP-coated acid sphingomyelinase (ASM) over free ASM in mouse models.

Specificity Index Tissues	Wildtype Mice	ASMKO Mice
Brain	3.05 ± 0.62	2.97 ± 0.65
Heart	1.89 ± 0.34	2.18 ± 0.56
Kidney	3.01 ± 0.47	3.22 ± 0.45
Liver	5.97 ± 1.0	2.89 ± 1.06
Lung	35.64 ± 4.02	33.63 ± 6.46
Spleen	26.25 ± 7.16	4.64 ± 4.43

Measurements refer to 30 min. Data are average ± SEM (*n* ≥ 3), where statistics were assessed by Student’s *t*-test (*p* < 0.05) to compare ASMKO vs. wildtype mice. None of the differences were significant.

## Data Availability

Data will be made available upon request.

## Supplementary Materials

The following supporting information can be downloaded at: https://www.mdpi.com/article/10.3390/ijms23074034/s1.
