# Supplementary material for: Comparison between Nanoparticle Encapsulation and Surface Loading for Lysosomal Enzyme Replacement Therapy
Source: Int J Mol Sci. 2022 Apr 6;23(7):4034. doi: 10.3390/ijms23074034 (PMC8999373; doi:10.3390/ijms23074034)
Supplement: Supplementary file 1 [file ijms-23-04034-s001.zip › ijms-1629955-supplementary(1).pdf]

Supplementary Information

Comparison between nanoparticle encapsulation and surface-loading for lysosomal enzyme replacement therapy

Eameema Muntimadugu [1], Marcelle Silva-Abreu [2], Guillem Vives [2], Maximilian Loeck [2], Vy Pham [1,3], Maria del Moral [2], Melani Solomon [1], and Silvia Muro [1,2,3,,4,*]

[1] Institute for Bioscience and Biotechnology Research, University of Maryland, College Park, MD, 20742, USA.
[2] Institute for Bioengineering of Catalonia of the Barcelona Institute for Science and Technology, Barcelona, 08028, Spain.
[3] Department of Chemical and Biomolecular Engineering, University of Maryland, College Park, MD, 20742, USA.
[4] Catalan Institution for Research and Advanced Studies, Barcelona, 08010, Spain.
* Correspondence: smuro@ibecbarcelona.eu.

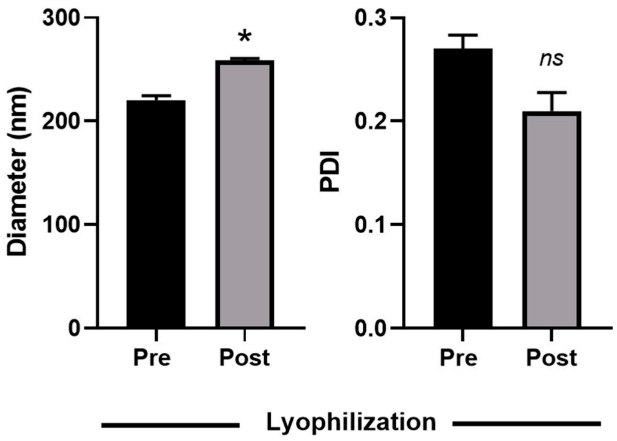

\

Figure S1. Effect of lyophilization on PLGA NPs encapsulating HAse. PLGA NPs prepared using Lactel II and Pluronic F68 and encapsulating HAse were directly characterized or subjected to lyophilization in the presence of 7.5% trehalose, then reconstituted and characterized. Their average hydrodynamic diameter and polydispersity index (PDI) are shown. Data are the mean ± SEM ($n \geq 3$), where statistics were assessed by Student's *t*-test ($p<0.05$) * Comparison of respective pre- vs. post-lyophilization parameters; ns No significant difference.

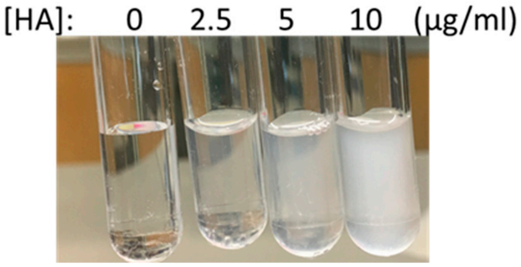

Figure S2. HA dependent turbidity. 1000-2200 kDa molecular weight hyaluronic acid (HA) was used as substrate to measure HAse activity in vitro. The picture shows the increase in turbidity of solutions containing increasing HA concentrations. On addition of HAse enzyme, HA would degrade, leading to reduced turbidity. Hence, by correlating the decreased HA absorbance upon incubation with HAse one can calculate the enzymatic activity.

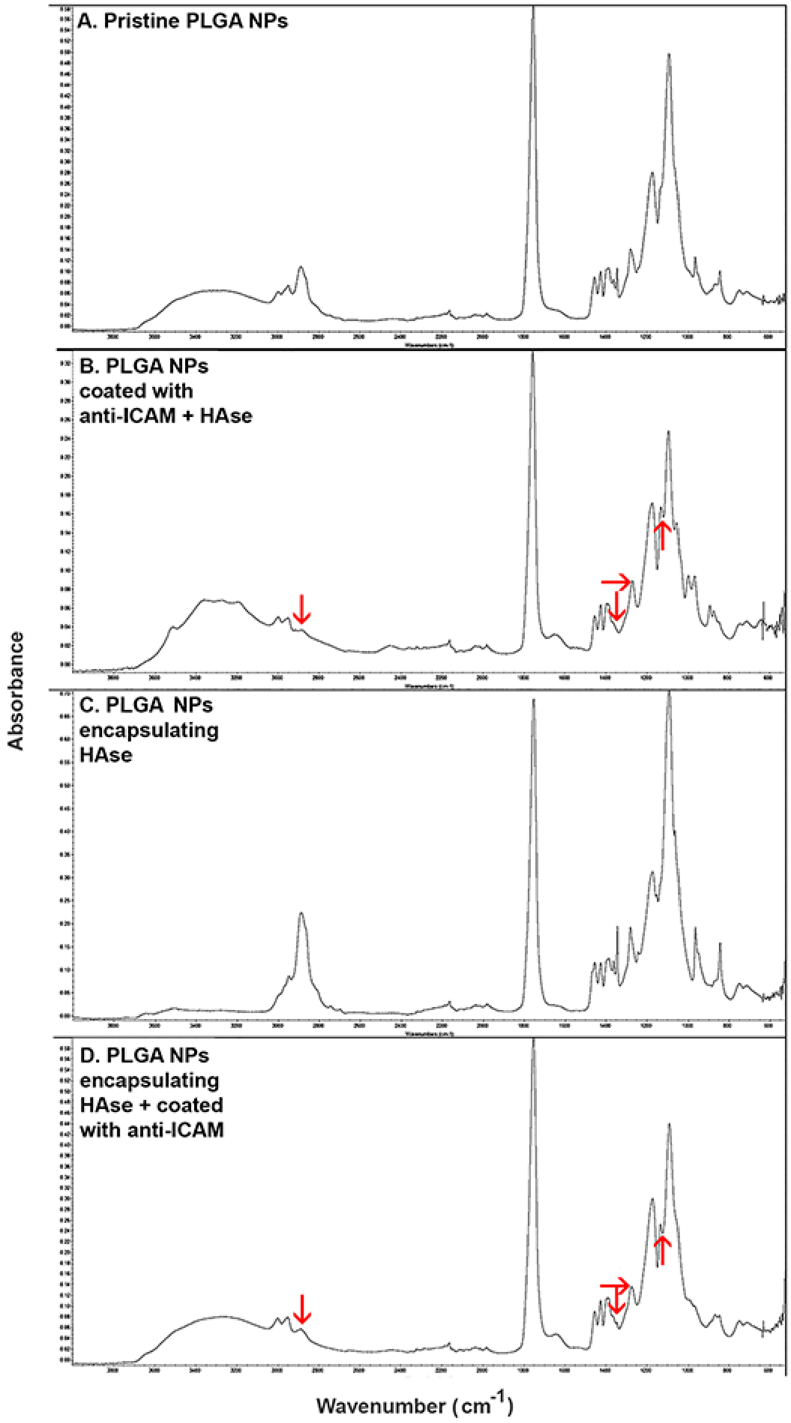

Figure S3. FTIR chatacterization of PLGA NPs. Fourier transform infrared (FTIR) spectra of (A) pristine PLGA NPs, (B) NPs coated with HAse and anti-ICAM, (C) PLGA NPs encapsulating HAse enzyme, and (D) NPs encapsulating HAse and coated with anti-ICAM. The spectra of non-coated formulations (A) and (C) were similar. Instead, coated formulations (B) and (D) had similar spectral alterations, e.g. peaks at 1343 cm^{-1} or 2886 cm^{-1} lowered or disappeared (↓), peak at 1278 cm^{-1} shifted to 1270 cm^{-1} (→), and peak at 1129 cm^{-1} appeared (↑).

Table S1. Characterization of enzyme-loaded anti-ICAM PLGA NPs.

NP formulation	Diameter (nm)	PDI	Enzyme molec./NP
Anti-ICAM/HAse NPs			
Coated formulations:	140.7 ± 10.9	0.15 ± 0.02	291.6 ± 54.1
Encapsulating formulations:	236.1 ± 30.2	0.21 ± 0.02	538.0 ± 68.0
Anti-ICAM/ASM NPs			
Coated formulations:	231.0 ± 15.0	0.24 ± 0.02	51.0 ± 3.0
Encapsulating formulations:	353.0 ± 3.0	0.20 ± 0.02	308 ± 58.0

Molec.=molecules. NP=nanoparticles. Data are the mean ± SEM (n=3)

Table S2. ^{125}**I-ASM biodistribution in wildtype mice expressed as %ID/g of tissue.**

Tissues:	Free ASM	ASM coated on NPs	ASM encapsulated in NPs
Blood			
1 min	39.6 ± 2.5	13.4 ± 1.6 *	9.2 ± 2.3 *
15 min	23.5 ± 1.7	4.8 ± 1.0 *	ND
30 min	20.9 ± 1.7	5.6 ± 0.9 *	1.0 ± 0.3 * $
Brain	0.56 ± 0.03	0.36 ± 0.1 *	0.14 ± 0.02 * $
Heart	3.2 ± 0.1	1.7 ± 0.3 *	2.5 ± 0.6
Kidney	7.2 ± 0.4	5.3 ± 0.4 *	4.3 ± 0.5 *
Liver	24.9 ± 1.3	35.8 ± 3.0 *	50.8 ± 5.6 * $
Lung	5.9 ± 0.7	59.9 ± 14.9 *	58.0 ± 14.3 *
Spleen	9.6 ± 0.6	53.8 ± 9.5 *	61.0 ± 7.0 *

*Organ measurements refer to 30 min. %ID/g=percentage of the injected dose per gram of tissue. ND=not detected. Data are average ± SEM (n≥4), where statistics were assessed by Student's t-test (p<0.05). * Compares either NP ASM vs. free ASM; $ Compares NP-encapsulated ASM vs. NP-coated ASM.*

Table S3. ^{125}I-ASM biodistribution in wildtype mice expressed as localization ratio.

Tissues:	Free ASM	ASM coated on NPs	ASM encapsulated in NPs
Brain	0.03 ± 0.001	0.08 ± 0.02 *	0.17 ± 0.04 * $
Heart	0.16 ± 0.01	0.30 ± 0.05	2.7 ± 0.6 * $
Kidney	0.35 ± 0.02	1.06 ± 0.17 *	5.1 ± 1.1 * $
Liver	1.24 ± 0.13	7.42 ± 1.24 *	62.1 ± 13.8 * $
Lung	0.28 ± 0.02	9.97 ± 1.12 *	68.2 ± 16.6 * $
Spleen	0.48 ± 0.04	12.54 ± 3.42 *	74.6 ± 21.4 * $

*Organ measurements refer to 30 min. Data are averages ± SEM (n≥4), where statistics were assessed by Student's t-test (p<0.05). * Compares either NP ASM vs. free ASM; $ Compares NP-encapsulated ASM vs. NP-coated ASM.*

Table S4. Specificity index for NP ^{125}I-ASM over free ^{125}I-ASM in wildtype mice.

Tissues	ASM coated on NPs	ASM encapsulated in NPs
Brain	3.0 ± 0.6	6.2 ± 0.001 *
Heart	1.9 ± 0.3	17.2 ± 0.002 *
Kidney	3.0 ± 0.5	14.4 ± 0.004 *
Liver	5.9 ± 1.0	49.9 ± 0.02 *
Lung	35.6 ± 4.2	244.0 ± 0.005 *
Spleen	26.2 ± 7.2	156.1 ± 0.01 *

*Organ measurements refer to 30 min. Data are average ± SEM (n≥4), where statistics were assessed by Student's t-test (p<0.05) to compares *NP encapsulated ASM vs. NPs coated ASM.*
